# Towards individualised treatment of urinary tract infections

**DOI:** 10.1038/s43856-025-00962-z

**Published:** 2025-07-01

**Authors:** Ellen V. Stadler, Alison Holmes, Danny O’Hare, Timothy M. Rawson

**Affiliations:** 1 Centre for Antimicrobial Optimisation, Imperial College London, London, UK; 2 Department of Infectious Diseases, Imperial College London, London, UK; 3 National Institute for Health Research, Health Protection Research Unit in Healthcare Associated Infections and Antimicrobial Resistance, Imperial College London, London, UK; 4 David Price Evans Infectious Diseases & Global Health Group, The University of Liverpool, Liverpool, UK; 5 The Fleming Initiative, Imperial College London and Imperial College Healthcare NHS Trust, London, UK; 6 Department of Bioengineering, Imperial College London, London, UK

**Keywords:** Prognostic markers, Bacterial infection

## Abstract

Stadler et al. propose using existing technologies to link urinary biomarkers and antimicrobial drug levels for personalised treatment of urinary tract infections. This approach aims to enable real-time pharmacokinetic-pharmacodynamic monitoring and optimise individual antibiotic dosing.

## Teaser

Monitoring host- and pathogen-specific biomarkers alongside drug levels at the site of infection offers promise for personalised antimicrobial therapy. Here, we highlight the potential of longitudinally monitoring biomarkers in urine using existing technologies to enable individualised PK-PD optimisation in urinary tract infections.

## Introduction

Antimicrobial drugs are used to treat bacterial infections and have revolutionised modern medicine. However, the inappropriate use and dosage of antimicrobials increases the risk of treatment failure, toxicity to the patient, and the development of drug resistance, rendering antimicrobials ineffective and putting medicine and global health at risk^1^. Bacterial urinary tract infection (UTI) affects 150 million people globally each year. On average, one in two adult women will experience a UTI during their lifetime^2^. Treating UTIs with empirical antibiotics when patients report symptoms results in overuse of antimicrobials, as more than half of the gold standard urine culture results of symptomatic females do not confirm a UTI^3^. Developing novel antimicrobials, enhancing diagnostic precision, and optimising the use of current treatments are approaches that can be used to mitigate the impact of drug resistance and maintain access to effective antimicrobial drugs^1^.

Both bacteria-specific and immune response biomarkers directly measured from the urine are frequently used to diagnose UTI, as the direct quantification of bacterial load in urine through culture is slow^4^. Their diagnostic value has been extensively explored, specifically compared to the gold standard culture and count. It is generally agreed that conventional biomarkers tested with urinary dipstick tests, such as nitrite and leucocyte esterase, have limited accuracy in the diagnosis of UTIs^5–7^. Only a few of these biomarkers have been explored longitudinally in the literature, such as neutrophil gelatinase-associated lipocalin or leucocyte esterase^8–10^. Similar to the diagnosis of UTIs through biomarker detection, the need for rapid susceptibility testing has also been addressed in the literature^11^. In vitro models mimic the urinary environment to understand the action and find the optimal dose of antimicrobial drugs^12^. But pharmacokinetic optimisation has not been explored in-vivo. Real-time monitoring of known biomarkers used for UTI diagnosis alongside drug concentration in urine has not yet been explored and could enable real-time estimation of treatment success.

In this Comment, we aim to present the unexplored potential of using current and emerging technologies to monitor biomarkers alongside urinary antimicrobial drug concentration in UTI to gain an understanding of the relationship between drug exposure linked with host and pathogen response. We argue this could be used towards real-time pharmacokinetic-pharmacodynamic (PK-PD) monitoring and support truly individualized approaches to the antimicrobial treatment of UTI.

## Challenges and opportunities in individualised antimicrobial dosing for urinary tract infections

There are significant variations in how antimicrobials are absorbed, distributed, metabolised, and excreted between patients (pharmacokinetics; PK). Additionally, the effect of an antimicrobial may vary based on organism and patient factors (pharmacodynamics; PD). Therapeutic drug monitoring (TDM) is a method of measuring drug concentration to achieve predefined PK-PD targets associated with clinical efficacy and to minimise the development of toxicity^13^. PD values, such as minimum inhibitory concentration (MIC), are assessed in-vitro or estimated using infection biomarkers such as C-reactive protein^14^. Delivery of individualised antimicrobial dosing through TDM is recommended in critical care settings for beta-lactam, glycopeptide, aminoglycoside, and oxazolidinone antibiotic classes^15^.

Individualised antimicrobial dosing using current TDM approaches can be challenging. Randomised clinical trials have shown no significant difference in mortality and microbiological cure rate between critically ill patients who undergo TDM and those who do not^16,17^. Important limitations of current approaches to TDM include the reliance on daily blood measurements of drug concentration and reliance on in-vitro PD values, such as MIC, that may not reflect drug concentration or antimicrobial PD at site of infection. To address these limitations, we need to consider technology for near real-time monitoring of individual PK-PD that can be performed closer to the site of infection and enable delivery of real-time dose adjustment.

UTI biomarkers are used for diagnosis, and the potential to use the temporal change of these biomarkers to monitor response to treatment in UTI has been discussed^18^ but not widely explored. TDM in urine has been reported to support dose optimisation in other infectious diagnoses, but not specifically for UTI management^19^.

## Supporting individualised dosing by quantifying host-bacteria-drug interactions

Adjusting antimicrobial dosing to improve PK-PD target attainment, using indices such as C_max_ to MIC ratio, results in improved treatment outcomes for patients with UTI^12^. Current PK-PD targets are based on population PK in serum and in-vitro MIC values. They do not consider variations in host factors such as immune response, site of infection, or local changes in pH, which affect both the infectious organism and the drug. For example, nitrofurantoin, a common first line treatment for UTI, demonstrates significantly increased bactericidal activity against common urinary pathogens at lower pH levels^20^. In-vivo PD estimation in urine has limited evidence of applicability to traditional antimicrobial susceptibility testing that is commonly used to guide antimicrobial selection^12^.

TDM has been used to optimise dosing in UTI by measuring drug concentrations in blood^21^, even though drug levels of antimicrobials in urine are typically considerably higher than in serum^22^. For example, amoxicillin, a beta-lactam antibiotic, has been demonstrated to achieve significantly greater peak urinary concentrations following standard dosing compared to serum. Local urinary concentration will often exceed traditional MIC breakpoints used for antimicrobial susceptibility reporting and may explain observed treatment success in the face of isolates that are reported as resistant^23^

The idea of connecting infection-specific biomarkers to antimicrobial PK to evaluate drug PD is well described. Galactomannan levels have been used to describe voriconazole PD in invasive aspergillosis in children^24^. Urinary biomarkers are frequently used to diagnose UTI, and the quantitative time-course of biomarkers, for example urinary leukocytes, has been shown to predict treatment outcome^10^.

We propose that the origin of well-described UTI biomarkers currently reported is either bacteria- or host-response-specific. Bacteria- and host-response biomarkers measured in urine could facilitate PD estimates for UTI if linked to observed drug exposure during treatment. This renders urine a promising area for the development of simple, non-invasive, longitudinal methods of monitoring and optimising antimicrobial PK-PD in real-time.

## The unexplored potential of longitudinal monitoring of drug exposure, host-response, and infection biomarkers in urine

Dipstick tests are frequently used to detect biomarkers qualitatively to support UTI diagnosis^25^. Below, we categorise biomarkers frequently used in dipstick tests, and recently described as promising for UTI diagnosis, into host-response and bacteria-specific biomarkers. The detected ranges of these biomarkers are shown in Table 1.

**Table 1:** Biomarker levels in non UTI and UTI patients

	Non-UTI			UTI		
	Mean	SD	Range	Mean	SD	Range
Nitrite^34^ [µM]	0.89	0.86	0.11–7.8	15.2	65	0.21–548
LE^38^ [U/L]	N/A	N/A	N/A	N/A	N/A	75–500
XO^31^ [U/L]	104.57	49.28	17–271	10,820.2	1543.7	5780–15,370
MPO^31^ [U/L]	414.09	93.31	156–745	1025.8	251.3	510–1546

Reported ranges of commonly used biomarkers for UTI diagnosis.

*LE* Leucocyte esterase, *XO* Xanthine Oxidase, *MPO* Myeloperoxidase.

Despite the development of quantitative point-of-care technologies for nitrite^18,26,27^, leucocyte esterase^28^ (LE), xanthine oxidase (XO)^29^ and myeloperoxidase (MPO)^30^ in urine, these biomarkers are frequently reported in a yes or no fashion. The sensitivity and specificity of XO and MPO for diagnosing UTIs show promising diagnostic values (100% and 87% sensitivity for XO and MPO, 100% specificity for both)^31^. Sensitivity and specificity of a positive nitrite and LE test vary depending on UTI prevalence and the bacterial count threshold used to define a UTI. Sensitivities in different studies range from 36–57% for nitrite and 72–95% for LE, and specificities 78–98% for nitrite and 9–59% for LE^32^.

**Fig. 1:**
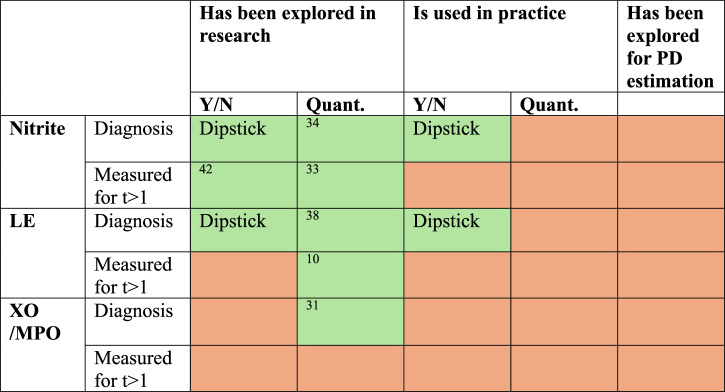
Overview of how UTI biomarkers are used in research and practice. UTI biomarkers are used for identification of UTIs, but their use for PD estimation is unexplored. Green: data available. Red: no data available. Y/N Qualitative, yes or no outcome, Quant Quantitative, LE Leucocyte esterase, XO Xanthine Oxidase, MPO Myeloperoxidase.

**Fig. 2:**
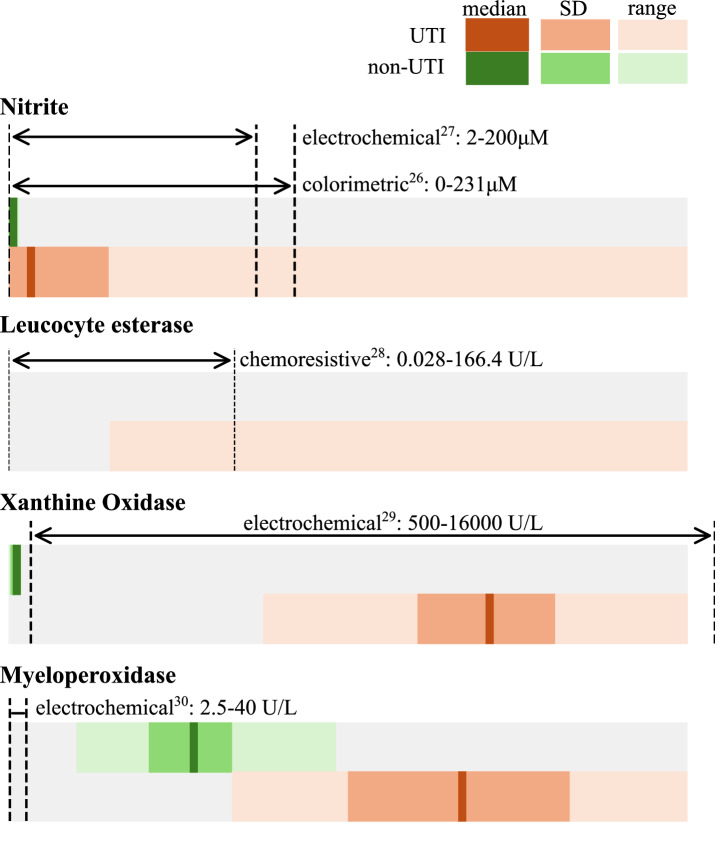
Detection range of point-of-care technologies and expected biomarker levels. Quantitative point-of-care sensors with detection range suitable to distinguish between UTI and non-UTI patients exist for Nitrite, Xanthine Oxidase and Myeloperoxidase. Length of bars corresponds to the range of values in Table 1. SD Standard deviation.

Few studies have evaluated biomarker concentration at more than one time point during UTI^10,33^, and the possibility of using quantitative measurements serially to estimate PD remains unexplored (Fig. 1). Longitudinal, quantitative monitoring of biomarkers in UTI using point-of-care technologies, alongside drug measurement, could facilitate novel approaches to real-time PK-PD monitoring and individualised antimicrobial dosing (Fig. 2).

Nitrite measurement is related to bacterial activity and is therefore a bacteria-specific biomarker. Maximum reported nitrite levels in UTI patients are 548 µM, and 7.8 µM in non-UTI patients^34^. Enterobacteriaceae, including *Escherichia coli*, which is responsible for most UTIs, use dietary nitrate as a secondary electron acceptor in oxygen-depleted urine and convert it to nitrite^35^. Limitations for nitrites use as an indicator of UTI include that nitrate to nitrite conversion can take up to 30 h to reach saturation, and not all bacteria that cause UTIs, such as *Enterococcus species* and *Staphylococcus saprophyticus*, exhibit nitrate-reducing activity^36^. Nitrite production over time in in-vitro broth culture of *Escherichia coli* and other bacteria has been explored^35^, but has not been assessed in patient samples.

Leucocyte esterase is an enzyme produced by neutrophils recruited during infection and is used as an indicator of urinary tract inflammation^37^. Leucocyte esterase can potentially indicate host response during UTI. The diagnostic value of nitrite and leucocyte varies between studies, which is believed to be due to different patient populations with varying pretest probabilities of a UTI^25^. Precise, sequential leucocyte esterase levels during antibiotic therapy in UTI can be used to predict antimicrobial outcome^10^, and should be explored in combination with bacteria-specific biomarkers such as nitrite. LE levels in UTI patients are reported up to 500 U/L^38^.

Xanthine Oxidase (XO) and Myeloperoxidase (MPO**)** have recently attracted attention as biomarkers exhibiting promising diagnostic properties for UTI^31^. XO is an enzyme responsible for the conversion of hypoxanthine to xanthine and uric acid. The origin of elevated XO during UTI is unclear but is likely a host-specific inflammation marker with mean levels of 10,820 U/L in UTI patients compared to 105 U/L in the urine of patients without UTI^39^. MPO is a host-specific biomarker with mean level of 1026 U/L observed in UTI samples compared to 414 U/L in non-UTI samples^31^.

## Concluding remarks

Sensitive, rapid, and inexpensive quantitative sensing technologies are needed for real-time PK-PD monitoring and dose adjustments. Various methods to detect nitrite quantitatively have been developed and tested in human urine, such as adaptation of the colorimetric Griess test for quantitative read-out^18,26^ or electrochemical detection^27^.

Whilst studies looking at detecting MPO and XO are sparse, as they are recently identified markers of UTI, proof-of-concept of their electrochemical detection in a point-of-care fashion to diagnose UTI exist^29,30^.

In the literature, the application of developed technologies to monitor UTI biomarkers longitudinally is discussed, but has not been directly explored. Table 1 summarises the observed range of organism and host-specific biomarkers in individuals with and without UTI. For nitrite, LE and XO, point-of-care technologies exist to quantitatively detect their concentration in the range of interest (Fig. 2). The electrochemical detection of MPO shows a narrow detection range (2.5–400 U/L)^40^, and the expansion of this range to monitor UTIs has not been discussed (Fig. 2). Drug concentration monitoring in UTI has been explored, but interestingly drug levels were assessed in serum and not in urine^21^.

To achieve truly individualised approaches to antimicrobial treatment of UTI, research must focus on evaluating longitudinal drug exposure and host- and organism-specific biomarkers measured in urine. Demonstrating the utility of longitudinal monitoring and linking drug exposure and host- and organism specific biomarkers to treatment outcome will support the development of point-of-care technologies for real-time antimicrobial optimisation for patients with UTI.

## References

[1] Rawson, T. M. et al. Optimizing antimicrobial use: challenges, advances and opportunities. *Nat. Rev. Microbiol.* **19**, 747–758 (2021). 10.1038/s41579-021-00578-9 34158654

[2] Yelin, I. et al. Personal clinical history predicts antibiotic resistance of urinary tract infections. *Nat. Med.* **25**, 1143–1152 (2019). 31273328 10.1038/s41591-019-0503-6 PMC6962525

[3] Butler, C. C. et al. Variations in presentation, management, and patient outcomes of urinary tract infection: a prospective four-country primary care observational cohort study. *Br. J. Gen. Pract.* **67**, e830–e841 (2017). 29158245 10.3399/bjgp17X693641 PMC5697553

[4] Karlsen, H. & Dong, T. Biomarkers of urinary tract infections: State of the art, and promising applications for rapid strip-based chemical sensors. *Analytical Methods* **7**, 7961–7975 (2015).

[5] Horváth, J., Wullt, B., Naber, K. G. & Köves, B. Biomarkers in urinary tract infections – which ones are suitable for diagnostics and follow-up? *GMS Infect. Dis.* **8**, Doc24 (2020). 33299741 10.3205/id000068 PMC7705555

[6] Edwards, G. et al. What is the diagnostic accuracy of novel urine biomarkers for urinary tract infection? *Biomarker Insights* **18**, 10.1177/11772719221144459 (2023). 10.1177/11772719221144459 PMC9902898 36761839

[7] Mattoo, T. K. & Spencer, J. D. Biomarkers for urinary tract infection: present and future perspectives. *Pediatric Nephrol.* **39** 2833–2844 (2024). 10.1007/s00467-024-06321-9 38483594

[8] Petrovic, S. et al. Clinical application neutrophil gelatinase-associated lipocalin and kidney injury molecule-1 as indicators of inflammation persistence and acute kidney injury in children with urinary tract infection. *Biomed. Res. Int.* **2013**, 947157 (2013). 23936859 10.1155/2013/947157 PMC3723056

[9] Hatipoglu, S. et al. Urinary MMP-9/NGAL complex in children with acute cystitis. *Pediatr. Nephrol.* **26**, 1263–1268 (2011). 21556719 10.1007/s00467-011-1856-3

[10] Ottiger, C., Schaer, G. & Huber, A. R. Time-course of quantitative urinary leukocytes and bacteria counts during antibiotic therapy in women with symptoms of urinary tract infection. *Clin. Chim. Acta* **379**, 36–41 (2007). 17229419 10.1016/j.cca.2006.11.023

[11] Davenport, M. et al. New and developing diagnostic technologies for urinary tract infections. *Nat. Rev. Urol.* **14** 298–310 (2017). 10.1038/nrurol.2017.20 PMC5473291 28248946

[12] Abbott, I. J., Roberts, J. A., Meletiadis, J. & Peleg, A. Y. Antimicrobial pharmacokinetics and preclinical in vitro models to support optimized treatment approaches for uncomplicated lower urinary tract infections. *Expert Rev. Anti Infective Therapy* **19** 271–295 (2021). 10.1080/14787210.2020.1813567 32820686

[13] Rawson, T. M. et al. Delivering precision antimicrobial therapy through closed-loop control systems. *J. Antimicrobial Chemother.* **73**, 835–843 (2018). 10.1093/jac/dkx458 PMC5890674 29211877

[14] Rawson, T. M. et al. Exploring the use of C-reactive protein to estimate the pharmacodynamics of vancomycin. *Ther. Drug Monit.* **40**, 315–321 (2018). 29561305 10.1097/FTD.0000000000000507 PMC6485622

[15] Abdul-Aziz, M. H. et al. Antimicrobial therapeutic drug monitoring in critically ill adult patients: a Position Paper#. *Intensive Care Med.* **46**, 1127–1153 (2020). 32383061 10.1007/s00134-020-06050-1 PMC7223855

[16] Ewoldt, T. M. J. et al. Model-informed precision dosing of beta-lactam antibiotics and ciprofloxacin in critically ill patients: a multicentre randomised clinical trial. *Intensive Care Med* **48**, 1760–1771 (2022). 36350354 10.1007/s00134-022-06921-9 PMC9645317

[17] Hagel, S. et al. Effect of therapeutic drug monitoring-based dose optimization of piperacillin/tazobactam on sepsis-related organ dysfunction in patients with sepsis: a randomized controlled trial. *Intensive Care Med.* **48**, 311–321 (2022). 35106617 10.1007/s00134-021-06609-6 PMC8866359

[18] Siu, V. S. et al. Toward a quantitative colorimeter for point-of-care nitrite detection. *ACS Omega* **7**, 11126–11134 (2022). 35415364 10.1021/acsomega.1c07205 PMC8991914

[19] Zentner, I. et al. Urine colorimetry for therapeutic drug monitoring of pyrazinamide during tuberculosis treatment. *Int. J. Infect. Dis.* **68**, 18–23 (2018). 29253711 10.1016/j.ijid.2017.12.017

[20] Fransen, F., Melchers, M. J. B., Lagarde, C. M. C., Meletiadis, J. & Mouton, J. W. Pharmacodynamics of nitrofurantoin at different pH levels against pathogens involved in urinary tract infections. *J. Antimicrobial Chemother.* **72**, 3366–3373 (2017). 10.1093/jac/dkx313 28961900

[21] Jayakumar, I., Mathaiyan, J., Mandal, J., Deepanjali, S. & Sreenivasan, S. K. Impact of therapeutic drug monitoring on once-daily regimen of amikacin in patients with urinary tract infection: a prospective observational study, www.drug-monitoring (2020). 10.1097/FTD.0000000000000800 32947556

[22] Wijma, R. A., Fransen, F., Muller, A. E. & Mouton, J. W. Optimizing dosing of nitrofurantoin from a PK/PD point of view: What do we need to know? *Drug Resistance Updates* **43** 1–9 (2019). 10.1016/j.drup.2019.03.001 30947111

[23] Levison, M. E. & Levison, J. H. Pharmacokinetics and pharmacodynamics of antibacterial agents. *Infect. Dis. Clin. N. Am.* **23**, 791–815 (2009). 10.1016/j.idc.2009.06.008 PMC3675903 19909885

[24] Huurneman, L. J. et al. Pharmacodynamics of voriconazole in children: Further steps along the path to true individualized therapy. *Antimicrob. Agents Chemother.* **60**, 2336–2342 (2016). 26833158 10.1128/AAC.03023-15 PMC4808208

[25] John, A. S., Boyd, J. C., Lowes, A. J. & Price, C. P. The use of urinary dipstick tests to exclude urinary tract infection. *Am. J. Clin. Pathol.* **126**, 428–436 (2006). 16880133 10.1309/C69RW1BT7E4QAFPV

[26] Noiphung, J. & Laiwattanapaisal, W. Multifunctional paper-based analytical device for in situ cultivation and screening of Escherichia coli infections. *Sci. Rep.* **9**, 1555 (2019). 30733495 10.1038/s41598-018-38159-1 PMC6367442

[27] Monteiro, T. et al. A quasi-reagentless point-of-care test for nitrite and unaffected by oxygen and cyanide. *Sci. Rep.* **9**, 2622 (2019). 30796298 10.1038/s41598-019-39209-y PMC6385495

[28] Tseng, W. T. et al. Quantitative urinary tract infection diagnosis of leukocyte esterase with a microfluidic paper-based device. *Dalton Trans.* **50**, 9417–9425 (2021). 34132300 10.1039/d1dt01541a

[29] Ding, X., Liu, X. & Lillehoj, P. B. Electrochemical detection in stacked paper networks. *J. Lab Autom.* **20**, 506–510 (2015). 25732354 10.1177/2211068215573662

[30] Hoyo, J., Bassegoda, A. & Tzanov, T. Electrochemical quantification of biomarker myeloperoxidase. *Z. fur Naturforsch. Sect. C. J. Biosci.* **77**, 297–302 (2022). 10.1515/znc-2021-0274 35191282

[31] Ciragil, P., Kurutas, E. B. & Miraloglu, M. New markers: Urine xanthine oxidase and myeloperoxidase in the early detection of urinary tract infection. *Dis. Markers* **2014**, 269362 (2014). 24591758 10.1155/2014/269362 PMC3925617

[32] Schmiemann, G., Kniehl, E., Gebhardt, K., Matejczyk, M. M. & Hummers-Pradier, E. Diagnose des harnwegsinfekts: Eine systematische übersicht. *Dtsch Arztebl* **107**, 361–367 (2010). 10.3238/arztebl.2010.0361 PMC2883276 20539810

[33] Smith, S. D., Wheeler, M. A., Lorber, M. I. & Weiss, R. M. Temporal changes of cytokines and nitric oxide products in urine from renal transplant patients. *Kidney Int.* **58**, 829–837 (2000). 10916108 10.1046/j.1523-1755.2000.00232.x

[34] Chao, M. R. et al. Urinary nitrite/nitrate ratio measured by isotope-dilution LC-MS/MS as a tool to screen for urinary tract infections. *Free Radic. Biol. Med.* **93**, 77–83 (2016). 26829019 10.1016/j.freeradbiomed.2016.01.025

[35] Tiso, M. & Schechter, A. N. Nitrate reduction to nitrite, nitric oxide and ammonia by gut bacteria under physiological conditions. *PLoS One* **10**, e0119712 (2015). 25803049 10.1371/journal.pone.0119712 PMC4372352

[36] Feng, S., Roseng, L. E. & Dong, T. Quantitative detection of Escherichia coli and measurement of urinary tract infection diagnosis possibility by use of a portable, handheld sensor. In *2015 IEEE International Symposium on Medical Measurements and Applications, MeMeA 2015 - Proceedings* 586–589 (Institute of Electrical and Electronics Engineers Inc., 2015). 10.1109/MeMeA.2015.7145271.

[37] Masajtis-Zagajewska, A. & Nowicki, M. New markers of urinary tract infection. *Clin. Chim. Acta* **471** 286–291 (2017). 10.1016/j.cca.2017.06.003 28622967

[38] Middelkoop, S. J. M., van Pelt, L. J., Kampinga, G. A., ter Maaten, J. C. & Stegeman, C. A. Routine tests and automated urinalysis in patients with suspected urinary tract infection at the ED. *Am. J. Emerg. Med.* **34**, 1528–1534 (2016). 27241566 10.1016/j.ajem.2016.05.005

[39] Giler, S., Henig, E. F., Urca, I., Sperling, O. & de Vries, A. Urine xanthine oxidase activity in urinary tract infection. *J. Clin. Pathol.* **31**, 444–446 (1978). 649770 10.1136/jcp.31.5.444 PMC1145301

[40] Bekhit, M. & Gorski, W. Electrochemical Assays and Immunoassays of the Myeloperoxidase/SCN - /H 2 O 2 System. *Anal. Chem.*10.1021/acs.analchem.8b05855 (2019). 30689356 10.1021/acs.analchem.8b05855

